# Vitamin B12 Deficiency in the Diagnostic Work-Up of Global Developmental Delay: A Treatable and Time-Sensitive Condition

**DOI:** 10.3390/nu18071098

**Published:** 2026-03-29

**Authors:** Rouzha Pancheva, Maria Dzhogova, Lyubomir Dimitrov, Miglena Nikolova, Galya Mihaylova, Veselina Panayotova, Diana A. Dobreva, Katya Peycheva, Bistra Galunska, Albena Merdzhanova

**Affiliations:** 1 Department of Hygiene and Epidemiology, Faculty of Public Health, Medical University of Varna “Prof. Dr. Paraskev Stoyanov”, 9002 Varna, Bulgaria; 2 Department of Biochemistry, Molecular Medicine and Nutrigenomics, Faculty of Pharmacy, Medical University of Varna “Prof. Dr. Paraskev Stoyanov”, 9002 Varna, Bulgaria; 3 Publishing Department, Medical University of Varna “Prof. Dr. Paraskev Stoyanov”, 9002 Varna, Bulgaria; ivanova@mu-varna.bg (V.P.); diana@mu-varna.bg (D.A.D.);; 4 Department of Chemistry, Faculty of Pharmacy, Medical University of Varna “Prof. Dr. Paraskev Stoyanov”, 9002 Varna, Bulgaria; albenamerdjanova@mu-varna.bg

**Keywords:** vitamin B12, cobalamin, global developmental delay, neurodevelopment, methylmalonic acid, holotranscobalamin, maternal nutrition

## Abstract

**Background:** Vitamin B12 deficiency is a recognized but frequently under-integrated cause of global developmental delay (GDD) in infancy and early childhood. Early diagnosis is critical because neurological impairment may be partially or completely reversible with timely treatment. **Objective:** This narrative review aims to synthesize current evidence on the role of vitamin B12 deficiency in the diagnostic evaluation of GDD, with a focus on clinical phenotype, risk factors, biomarkers, treatment outcomes, and practical integration into contemporary diagnostic algorithms. **Methods:** A structured, non-systematic search of PubMed/MEDLINE, Embase, and Web of Science was performed to identify clinical studies, case series, reviews, and guideline documents addressing pediatric vitamin B12 deficiency and neurodevelopmental delay. **Results:** Vitamin B12 deficiency in early childhood is most commonly associated with maternal deficiency and exclusive breastfeeding without adequate supplementation. Evidence from recent clinical and observational studies indicates that vitamin B12 deficiency may present with nonspecific neurological symptoms, including developmental regression, hypotonia, and feeding difficulties. Incorporating vitamin B12 assessment—using serum vitamin B12, holotranscobalamin, methylmalonic acid, and homocysteine—into early diagnostic algorithms for GDD may facilitate timely identification of a treatable cause of neurodevelopmental impairment. The proposed diagnostic framework emphasizes early biochemical evaluation in infants with unexplained developmental delay, thereby supporting prompt treatment during a critical window of neurological reversibility. **Conclusions:** Targeted assessment of vitamin B12 status in children with GDD, together with evaluation of maternal status, represents a clinically relevant approach to identifying a potentially preventable and treatable cause of neurodevelopmental impairment. Integration of functional biomarkers into diagnostic pathways and the development of pediatric-specific reference standards are key priorities for future research and clinical practice.

## 1. Introduction

Global developmental delay (GDD) is a common reason for referral to pediatric and neurodevelopmental services and is defined as a significant delay in at least two developmental domains in children younger than five years of age. Its etiology is heterogeneous and includes genetic and chromosomal abnormalities, structural brain malformations, perinatal insults, environmental exposures, and metabolic or nutritional disorders. Contemporary guidance recommends a structured, tiered diagnostic approach that maximizes diagnostic yield while prioritizing the identification of treatable causes [1,2,3].

Recent estimates suggest that GDD affects approximately 8.4% (95% CI: 7.7–9.1) of children under five years of age globally, corresponding to nearly 52.9 million affected children, with the greatest burden occurring in low- and middle-income countries [4,5]. Because many underlying causes are potentially preventable or treatable, early identification of modifiable risk factors remains a critical priority in pediatric healthcare.

Among potentially reversible conditions, vitamin B12 (cobalamin) deficiency is of particular clinical relevance. Vitamin B12 is an essential water-soluble vitamin required for one-carbon metabolism, DNA synthesis, mitochondrial function, and maintenance of myelin integrity. Its deficiency may lead to hematologic abnormalities and neurological manifestations, some of which may become irreversible if diagnosis and treatment are delayed. In infancy and early childhood, vitamin B12 deficiency most commonly reflects maternal deficiency—often related to vegetarian or vegan diets or malabsorption—although intrinsic absorption defects and inborn errors of cobalamin metabolism in the child must also be considered [6,7].

Affected infants and young children may present with hypotonia, apathy, feeding difficulties, failure to thrive, irritability, seizures, and developmental delay or regression. Hematologic findings such as macrocytosis or anemia are not universal, and their absence may delay recognition. Neuroimaging abnormalities, including cerebral atrophy and delayed myelination, have been described in severe cases. Evidence from case reports and small observational series suggests that timely vitamin B12 supplementation may result in rapid hematologic improvement and variable neurodevelopmental recovery, whereas delayed recognition is associated with persistent cognitive, motor, or behavioral sequelae [7,8,9,10].

Vitamin B12 deficiency in early childhood is therefore of particular clinical importance because it is both preventable and potentially reversible. However, within most diagnostic frameworks for GDD, it is mentioned only briefly among metabolic causes, without detailed consideration of its clinical phenotype, prognostic determinants, and practical integration into diagnostic algorithms [1,2,3]. Although the association between vitamin B12 deficiency and neurodevelopmental impairment has been documented previously, this review aims to provide an updated synthesis of recent clinical, biochemical, and neurodevelopmental evidence while emphasizing the structured integration of vitamin B12 assessment into the diagnostic work-up of children with GDD.

This narrative review aims to synthesize current evidence on vitamin B12 deficiency in the context of GDD, with three specific objectives: (1) to characterize the clinical and neuroimaging patterns associated with vitamin B12-related developmental delay and regression; (2) to examine the relationship between timing of diagnosis, duration of deficiency, and neurodevelopmental outcomes, including the concept of a potential window of reversibility; and (3) to provide practical, nutrition-oriented considerations for assessing vitamin B12 status in children with GDD and, when appropriate, in their mothers within contemporary diagnostic frameworks.

## 2. Materials and Methods

This article was conducted as a narrative review with a structured literature search focusing on vitamin B12 deficiency in the diagnostic work-up of global developmental delay (GDD) in infants and young children. The aim was to synthesize clinically relevant evidence rather than to perform a formal systematic review or meta-analysis.

### 2.1. Search Strategy

The literature search was performed in PubMed, Scopus, and Web of Science using combinations of the following keywords: “vitamin B12”, “cobalamin deficiency”, “global developmental delay”, “neurodevelopment”, “infant”, “biomarkers”, and “methylmalonic acid”. Search strings included combinations such as (“vitamin B12 deficiency” OR “cobalamin deficiency”) AND (“global developmental delay” OR “developmental regression” OR “neurodevelopment”). Titles and abstracts were initially screened for relevance, followed by full-text evaluation of potentially eligible articles. The screening process was conducted by the authors, and any disagreements regarding study inclusion were resolved through discussion.

### 2.2. Eligibility Criteria

The primary search focused on studies published between 2020 and 2026 in order to capture recent developments in biomarkers, neuroimaging findings, and clinical management of vitamin B12 deficiency in pediatric populations. Earlier landmark studies were also included when they provided foundational evidence regarding clinical presentation, neurodevelopmental outcomes, or treatment response.

The following types of publications were included:

- human studies reporting infants or children (generally ≤5 years of age) with documented vitamin B12 deficiency and associated neurodevelopmental delay, regression, or GDD;
- observational studies evaluating pediatric vitamin B12 status in relation to neurodevelopmental outcomes;
- case reports and case series describing clinical presentation, neuroimaging findings, and treatment response;
- position statements and guidelines on the evaluation of GDD that addressed metabolic or nutritional testing;
- narrative and systematic reviews relevant to vitamin B12 deficiency and neurodevelopment.

The following types of publications were excluded:

- adult-only studies;
- basic science or animal studies without direct clinical correlates;
- reports focusing exclusively on hematologic manifestations without description of neurodevelopmental outcomes.

Articles published in English were prioritized. Non-English studies with an English abstract were considered when they provided unique or clinically relevant data not otherwise represented in the literature.

### 2.3. Data Synthesis

Given the heterogeneity of study designs, sample sizes, and outcome measures, formal quality scoring and quantitative meta-analysis were not performed. Instead, the evidence was synthesized qualitatively and organized into predefined thematic domains corresponding to the structure of this review:

1. Vitamin B12 metabolism and its role in early brain development;
2. Epidemiology and risk factors for pediatric vitamin B12 deficiency;
3. Clinical presentation and neuroimaging patterns of vitamin B12-related developmental delay or regression;
4. Biomarkers of vitamin B12 status and diagnostic challenges;
5. Treatment response, timing of diagnosis, and long-term neurodevelopmental outcomes;
6. Integration of vitamin B12 assessment into contemporary diagnostic frameworks for GDD;
7. Limitations of the current evidence base and research gaps.

The synthesis aimed to identify consistent clinical patterns, areas of uncertainty, and practical implications for pediatric practice. During the preparation of this manuscript, the authors used ChatGPT (OpenAI, GPT-5.2) for language editing and structural refinement of the text. The authors critically reviewed and edited all generated content and take full responsibility for the accuracy and integrity of the final manuscript.

## 3. Narrative Synthesis

### 3.1. Vitamin B12 and Early Brain Development

Vitamin B12 (cobalamin) is essential for normal neurodevelopment, particularly during infancy, a period characterized by rapid myelination and synaptogenesis. Adequate vitamin B12 availability is required to sustain DNA synthesis, methylation reactions, and cellular energy metabolism in neurons and glial cells. Deficiency during this vulnerable developmental window may lead to structural and functional brain alterations, some of which may become irreversible if prolonged [11,12,13].

#### 3.1.1. One-Carbon Metabolism

**Figure 1:**
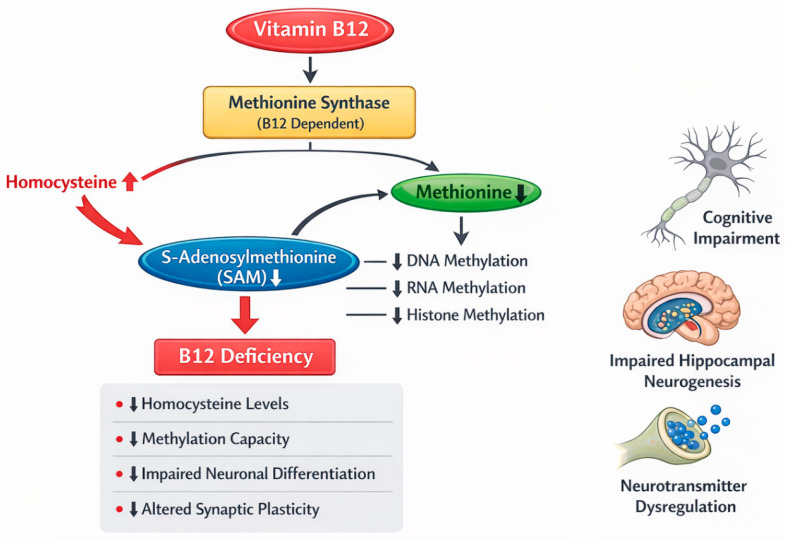
Disruption of vitamin B12–dependent methionine synthase activity and consequent alterations in SAM synthesis and neuronal function.

Vitamin B12 acts as a cofactor for methionine synthase, which catalyzes the remethylation of homocysteine to methionine and supports the synthesis of S-adenosylmethionine (SAM), the universal methyl donor for DNA, RNA, and protein methylation. Disruption of this pathway results in homocysteine accumulation and reduced methylation capacity, processes that are critical for neuronal differentiation, synaptic plasticity, and neurotransmitter synthesis [14,15,16]. Experimental models of maternal vitamin B12 restriction have demonstrated impaired hippocampal neurogenesis and long-term cognitive deficits in offspring, supporting the biological plausibility of vitamin B12-related neurodevelopmental vulnerability [15,17] (Figure 1).

#### 3.1.2. Myelination

**Figure 2:**
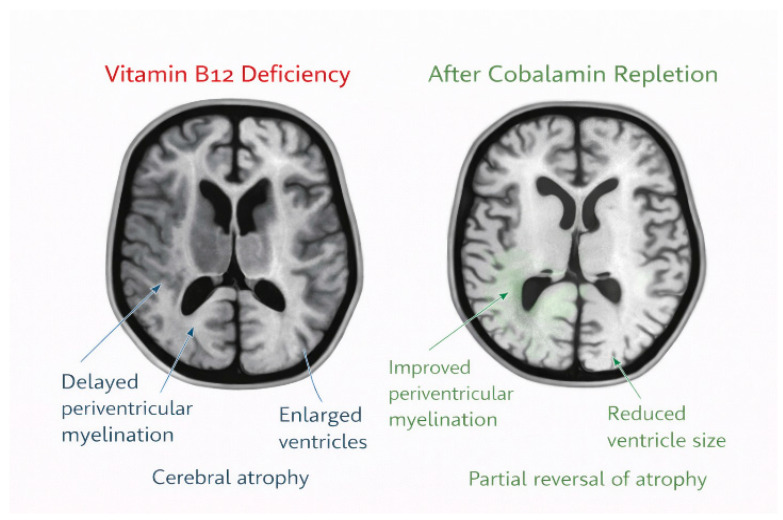
Brain MRI changes associated with severe infantile vitamin B12 deficiency and their potential improvement following cobalamin repletion.

**Figure 3:**
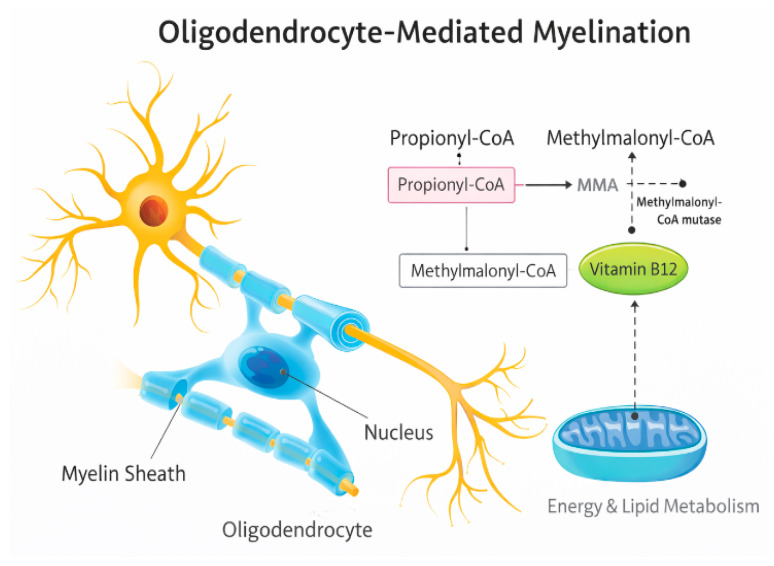
Proposed mechanism linking vitamin B12 deficiency with impaired myelination. Deficiency of vitamin B12 disrupts methylmalonyl-CoA mutase activity, leading to accumulation of methylmalonic acid (MMA), impaired lipid and energy metabolism, and altered oligodendrocyte function, which may contribute to defective myelin synthesis.

Vitamin B12 is also required for methylmalonyl-CoA mutase activity. In deficiency states, accumulation of methylmalonic acid may interfere with oligodendrocyte maturation and myelin lipid synthesis [12,18]. Clinically, severe infantile deficiency is associated with delayed myelination and cerebral atrophy on magnetic resonance imaging (MRI), particularly in the periventricular white matter. Figure 2 illustrates representative neuroimaging abnormalities reported in severe infantile vitamin B12 deficiency, whereas Figure 3 summarizes the upstream metabolic pathway linking vitamin B12 deficiency with impaired methylation (Figure 2 and Figure 3).

#### 3.1.3. Epigenetic Programming

Through its role in one-carbon metabolism, vitamin B12 contributes to the generation of methyl groups required for SAM-dependent methylation reactions during critical periods of brain development. DNA methylation is a key epigenetic mechanism regulating gene expression and influencing neuronal differentiation, synaptic plasticity, and long-term cognitive function. Low maternal vitamin B12 status has been associated with altered methylation patterns in offspring and with increased risk of neural tube defects and adverse neurocognitive outcomes [15,17]. Although these relationships are multifactorial, they support the concept that prolonged vitamin B12 insufficiency may have lasting effects on neurodevelopment beyond acute metabolic impairment.

#### 3.1.4. Mitochondrial Function

**Figure 4:**
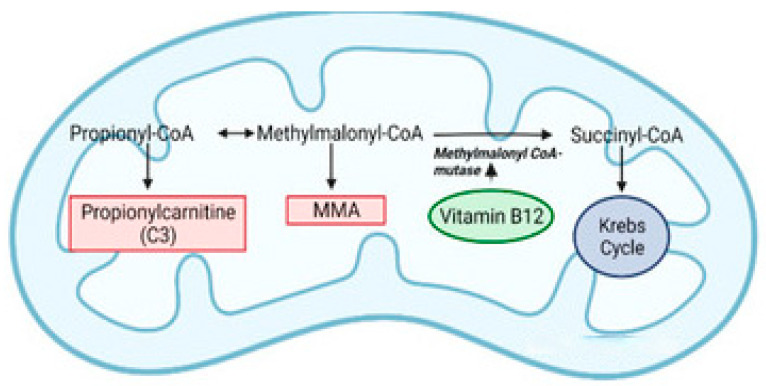
Vitamin B12-dependent methylmalonyl-CoA mutase reaction linking propionate metabolism to the tricarboxylic acid cycle.

Vitamin B12-dependent methylmalonyl-CoA mutase links propionate metabolism to the tricarboxylic acid cycle by generating succinyl-CoA. In cases of deficiency, accumulation of methylmalonic acid is associated with impaired mitochondrial respiration and increased oxidative stress, to which neurons are particularly vulnerable [12,18]. This metabolic disturbance likely contributes to the encephalopathy, hypotonia, and seizures observed in severe infantile deficiency. The rapid neurological improvement seen after parenteral treatment in many cases likely reflects restoration of cellular energy metabolism before irreversible neuronal injury occurs [18,19] (Figure 4).

## 4. Risk Factors for Pediatric Vitamin B12 Deficiency

Pediatric vitamin B12 deficiency results from a combination of maternal, dietary, gastrointestinal, genetic, and socioeconomic determinants. During infancy, vulnerability is heightened because neonatal stores are limited and rapid neurodevelopment increases metabolic demand [12,20,21,22].

### 4.1. Maternal Vitamin B12 Deficiency

Maternal vitamin B12 deficiency during pregnancy and lactation represents the most significant risk factor for infantile deficiency. Fetal stores depend on transplacental transfer, and breast milk cobalamin concentration closely reflects maternal status. Infants born to mothers with low vitamin B12 stores—due to pernicious anemia, prior gastric surgery, or inadequate dietary intake—are at high risk, particularly when exclusively breastfed [12,20,21,22]. Recent newborn screening and cohort data demonstrate that maternal vitamin B12 status is a strong predictor of infant status and that many severe infantile cases originate from previously unrecognized maternal insufficiency [20,21,22].

### 4.2. Exclusive Breastfeeding

Exclusive breastfeeding increases deficiency risk when maternal vitamin B12 levels are insufficient, as breast milk vitamin B12 content mirrors maternal circulating concentrations. Prolonged exclusive breastfeeding without timely introduction of complementary animal-source foods may lead to rapid depletion of infant stores [12,20,23,24,25]. Prospective data further indicate that delayed introduction of eggs or meat is associated with lower infant vitamin B12 status [23,24].

### 4.3. Vegetarian or Vegan Maternal Diet

Infants born to mothers adhering to vegetarian or, especially, vegan diets without adequate supplementation are at elevated risk, as vitamin B12 is found almost exclusively in animal-derived foods [22,24,26,27]. Case series and screening studies consistently report that vegan and macrobiotic dietary patterns during pregnancy and lactation are strongly associated with symptomatic infantile deficiency when supplementation is absent [22,24,27].

### 4.4. Malabsorption Syndromes

Pediatric vitamin B12 deficiency may also arise from malabsorptive disorders, including short-gut syndrome, inflammatory bowel disease, ileal resection, or congenital intrinsic factor deficiency. Maternal malabsorption—such as pernicious anemia or previous gastric bypass surgery—similarly increases infant risk by limiting fetal transfer and reducing breast milk vitamin B12 content [12,22,28].

### 4.5. Inborn Errors of Cobalamin Metabolism

Rare inherited disorders affecting vitamin B12 absorption (e.g., Imerslund–Gräsbeck syndrome), transport (e.g., transcobalamin II deficiency), or intracellular cobalamin processing and utilization (e.g., cblA, cblB, cblC, cblD, cblE, cblF, and cblG defects) can result in deficiency independent of dietary intake [28,29]. These conditions often present in early infancy with combined methylmalonic aciduria and homocystinuria and must be distinguished from nutritional deficiency because of their differing diagnostic and therapeutic implications.

### 4.6. Socioeconomic and Environmental Factors

Low socioeconomic status, limited access to animal-source foods, food insecurity, and lack of maternal supplementation during pregnancy are additional contributors to pediatric vitamin B12 insufficiency [22,26]. Early recognition of these risk factors is essential to prevent irreversible neurological sequelae.

## 5. Clinical Phenotype in Children with Global Developmental Delay

The clinical phenotype of GDD is heterogeneous and involves impairment across two or more developmental domains, including gross motor, fine motor, language, social-emotional, and adaptive functioning. A detailed developmental history is essential to distinguish developmental delay—defined as failure to achieve expected milestones—from developmental regression, characterized by the loss of previously acquired skills. This distinction is particularly relevant in the context of vitamin B12 deficiency, in which regression after a period of apparently normal development is a frequently reported pattern and should prompt urgent metabolic evaluation [30] (Table 1).

Neurological signs vary widely and may include hypotonia or hypertonia, abnormal deep tendon reflexes, movement disorders such as dystonia or ataxia, seizures, nystagmus, or focal neurological deficits. In vitamin B12 deficiency, hypotonia, apathy, developmental regression, and movement abnormalities are among the most consistently described findings. Abnormal head growth, although nonspecific, may reflect underlying brain atrophy in severe and prolonged deficiency. In contrast, dysmorphic features, neurocutaneous stigmata, or multiple congenital anomalies suggest alternative genetic or syndromic etiologies and may help differentiate nutritional causes from primary neurodevelopmental disorders [30,31,32].

Hematologic abnormalities are not defining features of GDD, but they have particular diagnostic value when present in children with neurodevelopmental symptoms. Macrocytosis or anemia, although not universal in vitamin B12 deficiency, may provide an important clue to an underlying nutritional or metabolic cause and should prompt targeted biochemical assessment [30].

Neuroimaging findings in GDD are variable and range from nonspecific abnormalities—such as reduced cerebral volume, delayed myelination, ventricular enlargement, or corpus callosum dysgenesis—to more specific malformations [31]. In vitamin B12 deficiency, delayed myelination and cerebral atrophy are the most commonly reported patterns, and their potential reversibility after treatment distinguishes this condition from many structural or genetic causes. Brain magnetic resonance imaging (MRI) is particularly indicated in children with developmental regression, abnormal tone, seizures, or altered head growth, as these features are frequently described in severe infantile deficiency [30].

Comorbidities frequently accompany GDD and may further refine the clinical phenotype. Feeding difficulties, failure to thrive, irritability, sleep disturbances, and behavioral changes are commonly reported in infants with vitamin B12 deficiency and often precede overt neurological signs. Recognition of this constellation—particularly when associated with exclusive breastfeeding, maternal dietary restriction, or other risk factors—can facilitate earlier diagnosis and treatment [30,31,32].

## 6. Biomarkers and Diagnostic Challenges

Biomarkers used to assess vitamin B12 status include total serum vitamin B12, holotranscobalamin (holoTC), methylmalonic acid (MMA), and homocysteine. Each marker reflects a different aspect of cobalamin metabolism, and no single test is sufficient in all clinical scenarios [12,33,34]. This has direct implications for the evaluation of children with GDD, in whom reliance on a single parameter may lead to missed diagnoses of a treatable condition (Table 2).

Total serum vitamin B12 remains the most widely used initial test because of its availability and cost-effectiveness. However, it has important limitations. It measures both the metabolically active fraction (holoTC-bound B12) and the inactive haptocorrin-bound fraction, resulting in limited sensitivity and specificity—particularly in the borderline range (approximately 180–350 pg/mL). In addition, falsely normal or elevated levels may occur in pernicious anemia because of assay interference from anti-intrinsic factor antibodies, as well as in liver disease and myeloproliferative disorders [12,33]. In the context of GDD, a “normal” total vitamin B12 concentration may therefore provide false reassurance and delay recognition of a potentially reversible cause.

HoloTC represents the biologically active fraction of circulating vitamin B12 that is available for cellular uptake. Several studies have demonstrated that holoTC has higher sensitivity than total vitamin B12 in detecting early or subclinical deficiency and may identify cases missed by conventional testing [34,35,36]. However, its routine use remains limited by cost, assay standardization, and variable availability across clinical laboratories, particularly in pediatric settings.

MMA is a functional biomarker that accumulates when vitamin B12-dependent methylmalonyl-CoA mutase activity is impaired. Elevated MMA is considered highly sensitive and relatively specific for vitamin B12 deficiency and is particularly useful in cases with borderline serum vitamin B12 concentrations [12,33,35,36,37]. Nonetheless, MMA levels may be falsely elevated in renal dysfunction, dehydration, and thyroid disorders, and age-dependent physiological variation complicates interpretation in infants and young children, for whom universally accepted pediatric reference ranges are still lacking.

Homocysteine concentrations increase in vitamin B12 deficiency because of impaired methionine synthase activity. Although sensitive, homocysteine is less specific than MMA because it is also elevated in folate deficiency, classic homocystinuria, renal failure, and other metabolic conditions [12,35,36]. Therefore, isolated elevation of homocysteine should be interpreted cautiously and in conjunction with other biochemical and clinical findings.

Functional vitamin B12 deficiency refers to a state in which metabolic markers (MMA and/or homocysteine) are elevated despite normal or borderline total serum vitamin B12 levels, suggesting impaired cellular utilization or transport. This phenomenon is increasingly recognized in pediatric populations and underscores the limitations of relying on total vitamin B12 alone when evaluating children with neurodevelopmental symptoms [34,36,37,38,39,40]. Composite indices, such as the combined indicator of vitamin B12 status (4cB12), integrate total vitamin B12, holoTC, MMA, and homocysteine into a single score and have been shown to improve diagnostic accuracy and reduce misclassification, particularly in borderline biochemical states [38,39]. Their clinical application in children remains limited, but they represent a promising approach for future pediatric diagnostic algorithms.

In summary, total serum vitamin B12 alone lacks adequate sensitivity and specificity for detecting early or functional deficiency. In children with GDD, assessment of functional biomarkers—particularly MMA and, where available, holoTC—reduces the risk of missing a treatable nutritional etiology. However, limited availability, cost, and the lack of standardized pediatric reference intervals remain important barriers to widespread implementation in routine clinical practice [12,33,34,35,36,37,38,39].

## 7. Treatment and Neurodevelopmental Outcomes

Vitamin B12 supplementation is the recommended treatment for children with confirmed deficiency presenting with GDD. Replacement therapy can be administered either orally or intramuscularly (IM), and both routes have been shown to effectively normalize biochemical markers and improve clinical outcomes, including hematologic and neurological manifestations [12,33,41,42].

Several clinical reports demonstrate that the neurological manifestations of vitamin B12 deficiency may improve rapidly following treatment. Classic case reports describe infants presenting with developmental regression, hypotonia, irritability, apathy, and feeding difficulties who exhibited marked neurological recovery after parenteral cobalamin replacement, often within weeks of therapy initiation [8,9,13]. These independent reports consistently describe rapid improvement in alertness, feeding behavior, and motor activity following treatment. In many cases, developmental skills lost during the period of deficiency were partially or completely regained after treatment [8,9,13]. These observations support the concept of a “window of reversibility,” during which prompt diagnosis and treatment may lead to substantial neurological recovery.

Intramuscular therapy is generally preferred in cases of severe deficiency, marked neurological involvement, suspected malabsorption, or when rapid correction is required. However, high-dose oral therapy (typically 1000–2000 µg daily) has been shown to be non-inferior to intramuscular administration in most cases, including pernicious anemia, and may offer advantages in cost, tolerability, and ease of administration [12,26,33,41,42,43]. IM therapy may remain necessary for children with significant neurological symptoms, poor adherence, or documented absorption disorders [26,33,43,44].

A hematologic response is usually observed within weeks, with reticulocytosis occurring early and correction of anemia typically achieved within 1–2 months after treatment initiation [12,45]. Neurological and developmental improvements often begin within weeks of therapy and may continue over several months. Clinical series and neuroimaging studies report substantial gains in developmental function by 3–6 months, with parallel improvements in MRI findings, including evidence of remyelination or reversal of cerebral atrophy in some cases [45,46,47].

Timely treatment is critical for optimizing neurodevelopmental outcomes. The likelihood of full recovery declines as the duration and severity of deficiency increase. Most infants and young children demonstrate significant improvement when treated promptly, whereas delayed diagnosis is associated with a higher risk of persistent cognitive, motor, or behavioral deficits [12,43,45,46,47]. The concept of a “window of reversibility” is particularly relevant in early infancy, when active myelination and synaptic development may still permit substantial recovery following correction of deficiency [45,46,47].

Observational data suggest that complete recovery may occur in approximately half of the treated patients, while most demonstrate at least partial improvement. Nonetheless, a subset—particularly those with prolonged or severe pre-treatment deficiency—may experience moderate to severe residual deficits despite adequate biochemical correction [45,46,47]. The duration and severity of symptoms prior to treatment initiation are among the strongest predictors of outcomes [44,45].

Several longitudinal observations further indicate that when diagnosis and treatment are substantially delayed, neurological recovery may be incomplete and residual cognitive or motor deficits may persist despite metabolic correction. Although neurological improvement following vitamin B12 therapy is often rapid, the degree of recovery depends strongly on the duration and severity of deficiency before treatment. Early intervention, particularly in infancy, may allow recovery of lost developmental milestones and normalization of developmental trajectories. In contrast, infants diagnosed only after the onset of neurological symptoms—such as developmental regression, hypotonia, seizures, or feeding difficulties—frequently present with more severe neurodevelopmental impairment. Long-term follow-up studies indicate that these children may exhibit persistent developmental delay, poor head growth, and residual neuroimaging abnormalities, including cerebral atrophy, thinning of the corpus callosum, and delayed myelination, even years after metabolic correction [12,48,49]. Although initial clinical improvement is commonly observed after vitamin B12 supplementation, only a minority of children diagnosed after the onset of neurological manifestations achieve normal developmental scores, while many remain within the borderline or mild disability range at long-term follow-up. These findings underscore the importance of early identification and treatment during the critical window of neurological reversibility, emphasizing that vitamin B12 deficiency represents a treatable but time-sensitive cause of GDD.

## 8. Integration of Vitamin B12 Assessment into the Diagnostic Work-Up of GDD

Maternal vitamin B12 status is a major determinant of infant vitamin B12 stores. Transplacental transfer during pregnancy and breast milk concentrations during lactation directly reflect maternal status. Consequently, infants born to mothers with low vitamin B12 levels—particularly those who are exclusively breastfed—are at heightened risk of deficiency [12,20]. Maternal deficiency may lead to inadequate fetal stores and low breast milk concentrations, resulting in symptomatic or subclinical deficiency in the child.

### 8.1. Indications for Testing

Assessment of vitamin B12 status should be considered in children presenting with GDD, particularly when risk factors are present. These include exclusive breastfeeding beyond early infancy without maternal supplementation, a maternal vegetarian or vegan diet, maternal malabsorptive disorders, or unexplained anemia in either the mother or the child [12,20,46].

Testing is also warranted in children with developmental regression, hypotonia, feeding difficulties, irritability, or unexplained neurological symptoms [12,20,46]. Because maternal deficiency is frequently the underlying cause of infantile vitamin B12 deficiency, evaluation of both the child and the mother is recommended when pediatric deficiency is suspected [20,21,22,24]. Universal screening of all children with GDD is not currently recommended; rather, testing should be guided by clinical features and risk profile [33].

### 8.2. Diagnostic Strategy

The diagnostic approach begins with measurement of total serum vitamin B12 in the child. If levels are borderline (approximately 180–350 pg/mL), or if clinical suspicion remains high despite a normal result, functional biomarkers—MMA and homocysteine—should be assessed to detect functional deficiency [12,20,33,36]. HoloTC may serve as an early and sensitive marker where available, although access remains limited in many clinical settings [36].

Combining biomarkers improves diagnostic performance. For example, low holoTC accompanied by macrocytosis or elevated MMA increases specificity for clinically meaningful deficiency [36]. Composite indices integrating multiple biomarkers may further enhance diagnostic accuracy, although their pediatric validation remains limited.

Newborn screening programs may identify some cases of maternal or infant vitamin B12 deficiency, but their sensitivity is incomplete, and they do not reliably detect all at-risk children—particularly those who develop deficiency later in infancy [21,24,50]. Therefore, clinical vigilance remains essential beyond the neonatal period.

### 8.3. Practical Implementation

**Figure 5:**
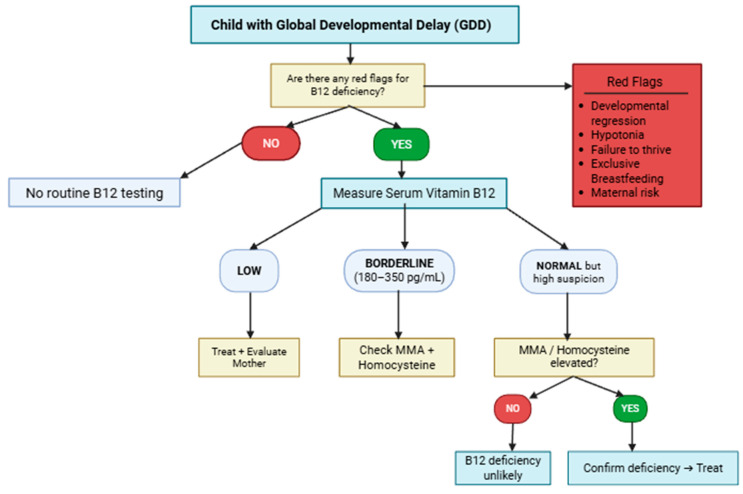
Algorithm for the evaluation of vitamin B12 deficiency in children with GDD.

A practical strategy for integrating vitamin B12 testing into the diagnostic work-up of GDD includes:

- obtaining a detailed dietary and feeding history from both the child and the mother;
- identifying maternal risk factors, including a vegetarian or vegan diet, previous bariatric surgery, and pernicious anemia;
- performing targeted laboratory testing for serum vitamin B12 and, when indicated, functional biomarkers;
- conducting parallel maternal evaluation when pediatric deficiency is suspected [Figure 5].

Early pregnancy screening of mothers at risk—particularly those with restrictive diets or malabsorptive conditions—may help prevent neonatal deficiency and subsequent neurodevelopmental complications [21,22,24]. Multidisciplinary follow-up is recommended to support treatment adherence and prevent recurrence in future pregnancies.

In resource-limited settings or high-prevalence populations, prioritizing testing in at-risk groups and incorporating vitamin B12 assessment into the structured evaluation of developmental delay may substantially improve early detection and intervention outcomes.

## 9. Limitations and Research Gaps

Despite increasing recognition of vitamin B12 deficiency as a reversible cause of neurodevelopmental delay, important limitations remain in both clinical practice and the current evidence base.

### 9.1. Heterogeneity of Clinical Presentation

The clinical phenotype of pediatric vitamin B12 deficiency is highly variable, ranging from subtle developmental delay to regression, hypotonia, feeding difficulties, irritability, seizures, and encephalopathy [12,46,48,51]. These manifestations overlap substantially with other etiologies of GDD, limiting the specificity of clinical assessment. Neuroimaging findings—such as cerebral atrophy, delayed myelination, and white matter changes—are supportive but not pathognomonic and cannot reliably distinguish vitamin B12 deficiency from other metabolic or neurodevelopmental disorders [12,48].

Most published data derive from case reports or small observational cohorts, often including heterogeneous age groups and variable severity, which limits generalizability and prevents precise phenotype stratification.

### 9.2. Uncertainty Regarding the Window of Reversibility

Although early treatment is consistently associated with improved outcomes, the precise duration of the “window of reversibility” remains insufficiently defined. Observational studies suggest that earlier intervention correlates with better neurodevelopmental recovery, whereas prolonged deficiency increases the risk of residual deficits [12,46,48,49,51]. However, most studies lack standardized developmental assessments, long-term follow-up, or control groups, limiting the ability to quantify recovery trajectories or identify definitive prognostic thresholds.

The proportion of children achieving complete versus partial recovery varies across reports, reflecting differences in study design, timing of diagnosis, and outcome measures [48,49]. Prospective longitudinal studies using standardized neurodevelopmental instruments are needed to clarify long-term cognitive and behavioral outcomes.

### 9.3. Diagnostic Limitations and Biomarker Uncertainty

Laboratory assessment presents additional challenges. Total serum vitamin B12 lacks optimal sensitivity and specificity, particularly in the borderline range, and may fail to detect functional deficiency [20,33,36,52]. Functional biomarkers—methylmalonic acid, homocysteine, and holoTC—improve diagnostic accuracy but are limited by availability, cost, and incomplete pediatric reference intervals [33,36,52].

Age-dependent variation in vitamin B12-related biomarkers further complicates interpretation in infants and young children [52]. Pediatric-specific reference ranges remain insufficiently standardized across laboratories and populations.

Newborn screening programs may detect some cases of deficiency, particularly those related to maternal status, but their sensitivity is incomplete, and many cases develop after the neonatal period [21,50]. Moreover, maternal screening strategies are not uniformly implemented, and there is no consensus regarding systematic assessment during pregnancy or lactation.

### 9.4. Gaps in Prevention and Screening Strategies

There is currently no standardized, evidence-based algorithm integrating maternal and infant screening within the evaluation of GDD. While targeted testing based on risk factors is recommended, the optimal balance between selective and broader screening approaches remains uncertain [21,36]. Further research is needed to:

- establish pediatric-specific reference intervals for functional biomarkers;
- define optimal biomarker combinations for early detection;
- determine long-term neurodevelopmental outcomes following treatment;
- evaluate the cost-effectiveness of maternal screening strategies;
- develop standardized diagnostic algorithms integrating maternal and infant assessment.

Emerging computational approaches may further improve the evaluation of neurodevelopmental consequences of vitamin B12 deficiency. In particular, advanced image analysis techniques, including deep learning-based methods for classification, detection, and segmentation, may enable more precise quantification of neuroimaging changes such as brain volume, cortical thickness, or myelination patterns over time. Although these methods have primarily been developed in other imaging fields, they may in the future support more objective monitoring of treatment responses in pediatric neurodevelopmental disorders.

## 10. Conclusions

Vitamin B12 deficiency is a recognized and treatable cause of GDD in infancy and early childhood. Because clinical manifestations are heterogeneous and may occur without overt hematologic abnormalities, laboratory assessment is essential when risk factors or suggestive neurological features are present. Maternal status plays a central role, particularly in exclusively breastfed infants and in families with limited animal-source food intake.

Serum vitamin B12 remains the initial test, but functional biomarkers such as methylmalonic acid and homocysteine improve detection of early or borderline deficiency. Prompt treatment is associated with substantial neurodevelopmental improvement, whereas delayed recognition increases the risk of persistent deficits.

Integrating targeted vitamin B12 testing—alongside maternal evaluation—into the structured diagnostic work-up of children with GDD may help prevent irreversible neurological outcomes. Further research is needed to standardize pediatric biomarker thresholds, validate diagnostic algorithms, and optimize screening strategies.

## Figures and Tables

**Table 1:** Clinical clues that may help differentiate vitamin B12-related GDD from genetic or syndromic causes of GDD.

Clinical Domain	B12-Suggestive Phenotype	Genetic/Syndromic Phenotype
**Developmental pattern**	Developmental regression after a period of apparently normal development; loss of previously acquired skills	Persistent developmental delay; multiple congenital anomalies may suggest a syndromic cause
**Neurological signs**	Hypotonia; apathy; movement abnormalities (e.g., dystonia, ataxia); seizures; abnormal deep tendon reflexes; nystagmus; focal neurological deficits	Dysmorphic features; neurocutaneous stigmata; multiple congenital anomalies
**Head growth**	Abnormal head growth may reflect cerebral atrophy in severe and prolonged deficiency	Congenital anomalies may indicate a genetic or syndromic etiology
**Hematologic findings**	Macrocytosis; anemia (not universal, but diagnostically informative)	Not typically a defining feature
**Neuroimaging findings**	Delayed myelination; cerebral atrophy; potential reversibility after treatment	Structural malformations; corpus callosum dysgenesis; more specific congenital abnormalities
**Associated clinical features**	Feeding difficulties; failure to thrive; irritability; sleep disturbances; behavioral changes; association with exclusive breastfeeding or maternal dietary restriction	Not specifically associated with nutritional risk factors

**Table 2:** Biomarkers used to assess vitamin B12 status in children with GDD.

Biomarker	Biological Role/What It Reflects	Strengths	Limitations/Diagnostic Challenges
**Total Serum Vitamin B12**	Total circulating vitamin B12 (active + inactive fractions)	Widely available; cost-effective; standard initial screening test	Limited sensitivity, especially in the borderline range (180–350 pg/mL); measures inactive (haptocorrin-bound) fraction; false normal/elevated levels in pernicious anemia due to anti-intrinsic factor interference; affected by liver disease and myeloproliferative disorders; cannot detect functional deficiency
**Holotranscobalamin (holoTC)**	Biologically active transcobalamin-bound fraction of vitamin B12 available for cellular uptake	Early marker of deficiency; higher sensitivity than total vitamin B12 in early/subclinical states	Limited availability; higher cost; incomplete pediatric standardization; variable laboratory implementation
**Methylmalonic Acid (MMA)**	Functional intracellular deficiency due to impaired methylmalonyl-CoA mutase activity	High sensitivity; relatively specific for vitamin B12 deficiency; particularly useful in borderline total vitamin B12 levels	Elevated in renal dysfunction; affected by dehydration and thyroid disorders; age-dependent physiological variation in infants; lack of universally standardized pediatric reference ranges
**Homocysteine**	Impaired methionine synthase activity secondary to vitamin B12 deficiency	Sensitive marker; reflects functional metabolic impairment	Low specificity; elevated in folate deficiency, renal disease, and inherited metabolic disorders (e.g., homocystinuria)

## Data Availability

No new data were created or analyzed in this study. Data sharing is not applicable to this article.

## Abbreviations

The following abbreviations are used in this manuscript:

GDD	global developmental delay
SAM	S-adenosylmethionine
holoTC	holotranscobalamin
MMA	methylmalonic acid
IM	intramuscular
